# A tool to facilitate clinical biomarker studies - a tissue dictionary based on the Human Protein Atlas

**DOI:** 10.1186/1741-7015-10-103

**Published:** 2012-09-12

**Authors:** Caroline Kampf, Julia Bergman, Per Oksvold, Anna Asplund, Sanjay Navani, Mikaela Wiking, Emma Lundberg, Mathias Uhlén, Fredrik Ponten

**Affiliations:** 1 Department of Immunology, Genetics, Pathology, Science for Life Laboratory, Uppsala University, Dag Hammarskjölds väg 20, Uppsala, 751 85 Sweden; 2 Science for Life Laboratory, Royal Institute of Technology, Tomtebodavägen 23A, Solna, 171 65, Sweden; 3 Lab Surgpath, 204 Bombay Market, Tardeo Main Road, Mumbai, 400 034, India

**Keywords:** Antibody-based proteomics, cancer biomarkers, tissue and cell dictionary, immunohistochemistry, protein expression, histology, pathology

## Abstract

The complexity of tissue and the alterations that distinguish normal from cancer remain a challenge for translating results from tumor biological studies into clinical medicine. This has generated an unmet need to exploit the findings from studies based on cell lines and model organisms to develop, validate and clinically apply novel diagnostic, prognostic and treatment predictive markers. As one step to meet this challenge, the Human Protein Atlas project has been set up to produce antibodies towards human protein targets corresponding to all human protein coding genes and to map protein expression in normal human tissues, cancer and cells. Here, we present a dictionary based on microscopy images created as an amendment to the Human Protein Atlas. The aim of the dictionary is to facilitate the interpretation and use of the image-based data available in the Human Protein Atlas, but also to serve as a tool for training and understanding tissue histology, pathology and cell biology. The dictionary contains three main parts, normal tissues, cancer tissues and cells, and is based on high-resolution images at different magnifications of full tissue sections stained with H & E. The cell atlas is centered on immunofluorescence and confocal microscopy images, using different color channels to highlight the organelle structure of a cell. Here, we explain how this dictionary can be used as a tool to aid clinicians and scientists in understanding the use of tissue histology and cancer pathology in diagnostics and biomarker studies.

## Background

The Human Protein Atlas project, launched in 2003, was initiated as a natural extension of the Human Genome Project, with the objective to explore the proteins encoded by the human genome. The primary focus was to analyze the distribution and relative abundance of all proteins in human normal cells and tissues, and to determine the subcellular localization of each protein. One main goal in this effort was to contribute to biomedical and clinical research, and because cancer is a major disease where diagnostics, classification and prognostic stratification is based on tissue morphology, a multitude of clinical cancer tissue samples were included in the comprehensive protein profiling. This has allowed researchers to utilize the protein profiling data for both biomarker discovery efforts and for validation of altered gene expression patterns at the protein level in both normal and cancer tissue.

The Human Protein Atlas project pursues a systematic high-throughput generation of affinity-purified polyclonal antibodies with the aim of generating a map of protein expression patterns on a proteome-wide scale in both human normal cells, tissues and organs, and in cancer tissues [1]. Immunohistochemistry (IHC) is performed on tissue micro arrays (TMA), containing a multitude of different normal tissues and tumors, to enable a comprehensive mapping of protein expression patterns at cellular resolution in a tissue context. Altogether 144 different normal tissues are analyzed together with 216 different tumors representing the 20 most common forms of human cancer [2]. Immunofluorescence (IF)-based profiling of protein expression in cell lines is performed to generate a map of subcellular localization patterns [3]. All protein expression data, including the underlying images, are made publicly available at the Human Protein Atlas web portal (http://www.proteinatlas.org) [4]. The current version of the Human Protein Atlas contains data for more than 14,000 unique proteins. This corresponds to more than 70% of all human protein encoding genes [5].

As the cell constitutes the smallest living entity, it is required to harbor specialized and distinct subcellular structures. Cells vary considerably in function and morphology and these differences form the basis for the concept of different cellular phenotypes. On a higher level, cell types with their distinct phenotypes are organized into tissues, commonly categorized as epithelial, muscle, vascular, nervous and connective tissue, and hematopoietic cells. Genetic changes leading to dysregulated signaling pathways with altered protein expression patterns cause a transformation from normal to the phenotypes and morphology that signifies cancer. Cancer is a heterogeneous disease associated with alterations in protein expression patterns leading to cell growth and 'anti-social behavior' of tumor cells. The deregulated expression patterns in tumor cells are caused by genetic and epigenetic alterations leading to distortion of multiple proteins and signaling pathways. Despite the complexity of cancer, microscopic evaluation of tissue morphology remains the gold standard for determining a cancer diagnosis in a clinical setting. Although morphology is crucial, adding a layer of information regarding protein expression on top of morphology appears to be beneficial for the stratification of different tumor types. Immunohistochemistry prevails as an invaluable method to provide such a tool for visualization of protein expression patterns in cells from a section of tumor tissue.

## The Dictionary - a tool for biomarker studies

**Figure 1:**
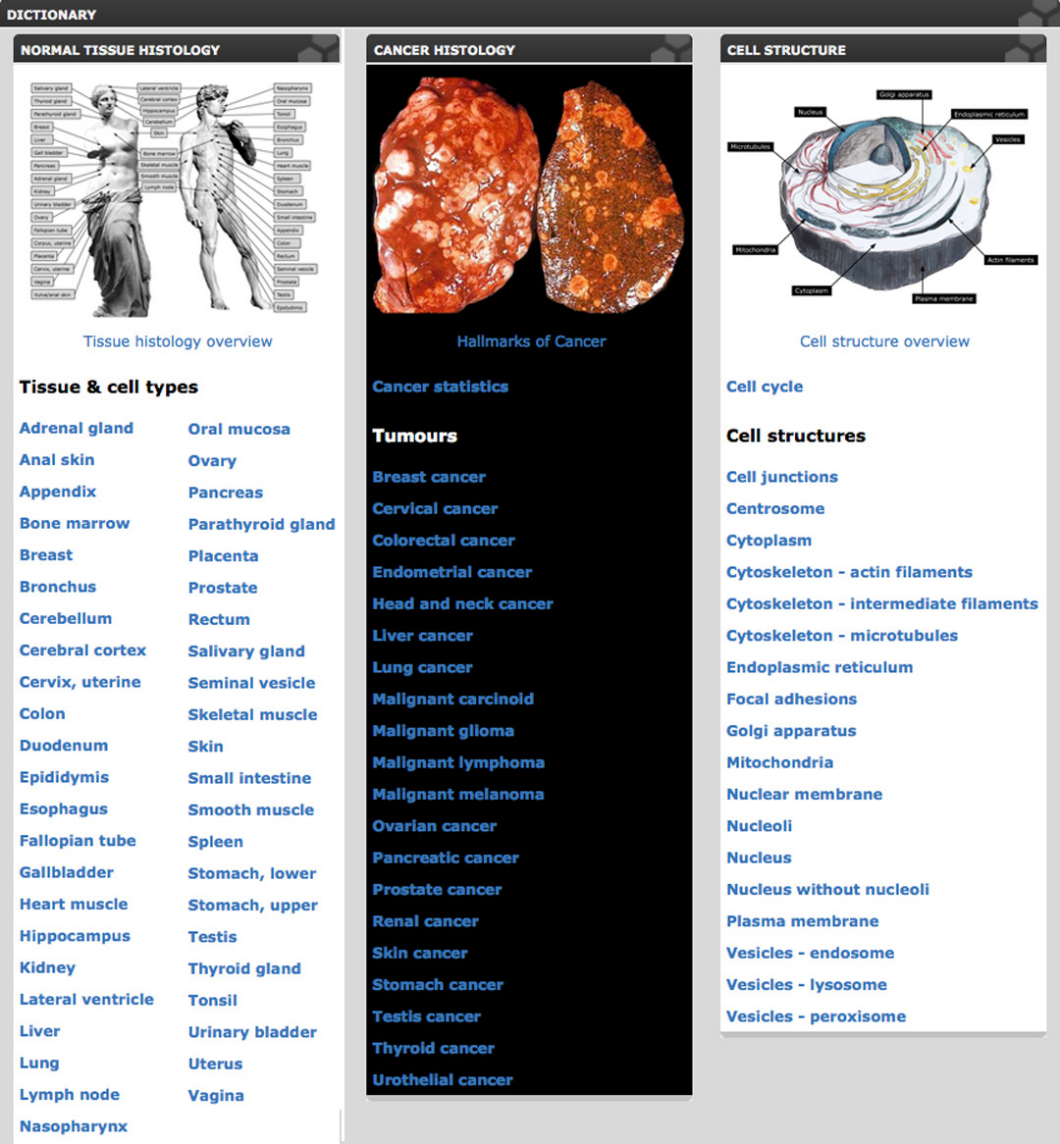
**Schematic showing the starting page for the dictionary**. The three main parts, normal tissues, cancer tissues and cell structures, are displayed side by side with alphabetical lists below showing the contents of each part to facilitate navigation. All figures are original and available at the Human Protein Atlas web portal (www.proteinatlas.org/dictionary). Published with permission from the Human Protein Atlas.

The dictionary contains three main parts: normal tissues, cancer tissues, and cells (http://www.proteinatlas.org/dictionary) (Figure 1). All images and examples include descriptive text boxes and supporting text with background information, to facilitate interpretation of the complex patterns underlying normal tissue histology, tumor pathology and cell biology. H & E-stained tissue sections have been scanned at 40 × magnification and both normal and cancer tissues are shown at three different levels of magnification.

**Figure 2:**
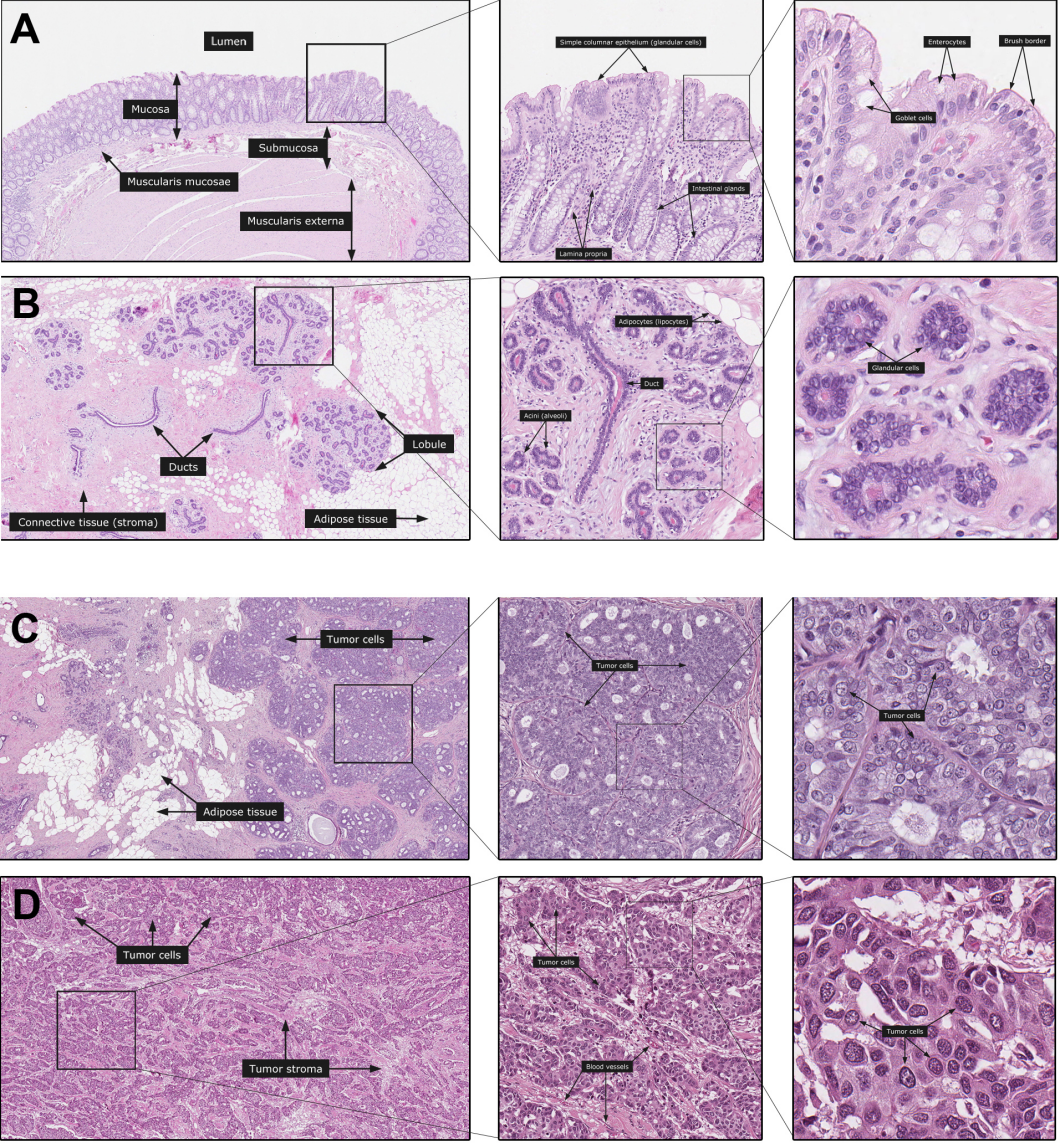
**Images showing examples of H & E-stained tissues, including descriptive text boxes, at three different magnifications**. Normal tissue is exemplified by two tissue types. The top overview shows the major components of a normal human colon, followed by higher magnifications revealing the glandular structure of the mucosa with regularly ordered colonic crypts. The finer details of integrated cells and structures are apparent at the highest level of magnification (**A**). The overview of a normal female breast shows the arrangement of included normal glandular lobules and ducts with magnifications showing a more detailed view of a single lobular unit and details of glandular cells (**B**). Cancer is exemplified by two cases of breast cancer. A ductal breast carcinoma with low grade malignancy (Elston-Ellis score 4) from a female patient age 68 is displayed at three levels of magnification to demonstrate the overall pattern of tumor growth and the finer details of cancerous glands and details of cancer cells (**C**). A high grade (Elston-Ellis score 9) ductal breast carcinoma from a female patient age 83 shows the characteristics of infiltrative tumor growth with poorly differentiated glandular structures and severe cellular atypia (**D**). All figures are original and available at the Human Protein Atlas web portal (www.proteinatlas.org/dictionary). Published with permission from the Human Protein Atlas.

**Figure 3:**
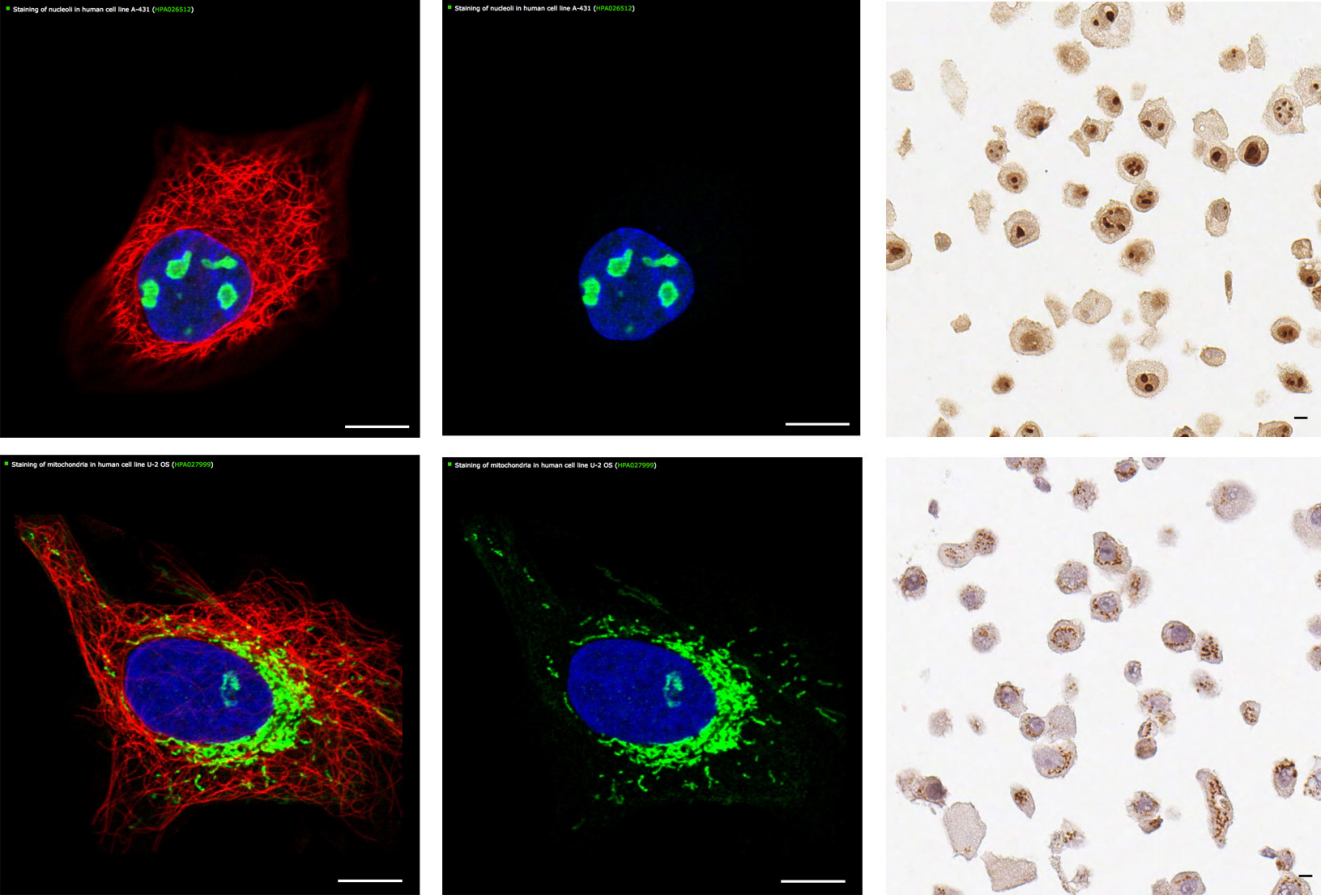
**Examples of images demonstrating different organelles in cells**. The upper panel shows IF (left and middle) and IHC (right) images representing the nucleoli, visualized by antibodies targeting proteins expressed in the nucleoli. The nucleoli are shown as a green color in the IF example and brown color in the IHC example. The lower panel shows images representing mitochondria, visualized by antibodies expressed in mitochondrion. IF: green - antibody (HPA026512, HPA027999); blue - nucleus (DAPI), red - microtubule. IHC: brown - antibody (HPA005768, HPA004016). Scalebar 10 μm. IF, immunofluorescence; IHC, immunohistochemistry. All figures are original and available at the Human Protein Atlas web portal (www.proteinatlas.org/dictionary). Published with permission from the Human Protein Atlas.

Altogether 45 normal tissue types (represented by 173 images), 20 different cancer types (represented by 193 images) and 18 subcellular structures (represented by 103 images) are included in the dictionary. Examples of normal tissue show colon (Figure 2A) and breast (Figure 2B) at the three levels of magnification. For cancer, one case of low-grade (Figure 2C) and one case of high-grade (Figure 2D) ductal breast cancer is shown. IF and IHC images representing antibodies targeting proteins in the nucleoli and the mitochondria demonstrate the cell structure part (Figure 3). In addition to high resolution images, there are summarizing descriptive text paragraphs to supplement the images.

As one of the main goals of this project is to identify novel biomarkers that can be developed for clinical use, the 20 types of human cancers illustrated in the dictionary have also been used for protein profiling in the Human Protein Atlas. Using the search function at the Human Protein Atlas portal [6], search strings can be created to identify candidates for cell or tumor type specific markers and also proteins differentially expressed within a given tumor type, thus representing potential prognostic indicators.

## Clinical impact

Successful identification and translation of informative biomarkers to aid clinical decision-making is a prerequisite for the implementation of personalized cancer therapeutic regimens. The antibody-based proteomics strategy employed in the Human Protein Atlas plays a key role in the cancer biomarker discovery and validation pipeline, facilitating evaluation of candidate markers [7]. The newly launched dictionary provides a useful tool to interpret and evaluate biomarker candidates identified through various search strategies in the Human Protein Atlas. The appraisal of protein expression patterns in tumor tissue is a crucial step to select the most promising candidates for extended experiments, including clinical studies in larger cohorts, functional studies and in-depth validation of expression patterns.

The Human Protein Atlas has already been used in several clinical biomarker studies as a starting point for exploring both diagnostic and prognostic factors. Cell and tumor type specific protein expression, critical for developing diagnostic markers, is exceedingly rare [8], and only a few such markers exist for clinical use. As an example, the DNA-binding protein SATB2 was identified in the Human Protein Atlas as a potential novel diagnostic marker for colorectal cancer and in an extended study including more than 2,400 tumors, SATB2 was found to be both a sensitive and highly specific marker for colorectal cancer [9]. The basic protein profiling data available in the Human Protein Atlas has also allowed for several potential prognostic cancer biomarkers to be identified for different types of cancer. This is exemplified by the RNA-binding protein RBM3, discovered to be a prognostic marker for several different forms of cancer [10-12], and also a potential treatment predictive marker for platinum-based therapies [13]. Understanding of tumor tissue composition is also fundamental for studies regarding tumor stroma compartments. In a recent tumor biology study [14] using a mouse model, large numbers of granulin-expressing bone marrow-derived hematopoietic cells were found in the tumor stroma of breast cancers responding to instigating signals. This study also showed that the expression of granulin in human breast cancer was strongly correlated with the triple negative/basal-like breast tumor subtypes, and that breast cancer patients with tumors positive for granulin staining had a significantly worse outcome in terms of overall survival. The presented dictionary and Human Protein Atlas can also be utilized in other clinical research fields, exemplified by the identification of targets for *in vivo *imaging of pancreatic beta-cells cells in diabetes research [15,16].

## Concluding remarks

Here we present a freely available cell and tissue dictionary as an amendment to the Human Protein Atlas (reviewed in [17]) that can be used to facilitate the interpretation of clinical tissue biomarkers. A multitude of high quality images with supporting short text paragraphs are displayed on the Human Protein Atlas web portal (http://www.proteinatlas.org/dictionary) to provide a useful guide for researchers who are not familiar with the microscopical landscape that forms the home ground for histologists and pathologists. In this first version of the dictionary, H & E stained tissue sections are presented for visualization of normal tissue and cancer morphology. The essential background needed to interpret and understand expression data retrieved from tissues and cells is presented. The aim is to expand the contents of the dictionary to also include additional levels of information regarding protein expression so that various cell populations not distinguishable from morphology alone can be visualized. Established antibodies can be used for IHC on consecutive sections of selected tissues to demonstrate different cell types, for example, B-lymphocytes, T-lymphocytes and endothelial cells, and different cell states, for example, proliferation and differentiation. Moreover, updates with additional links and text paragraphs can be added as well as including more examples of both normal and diseased tissues. For educational purposes, show/hide functionality for text boxes could be further developed together with sets of relevant 'Questions & Answers'. We anticipate that a content rich and knowledge-based cell and tissue dictionary, combined with the comprehensive map of protein expression patterns in normal and cancer tissues available through the Human Protein Atlas, will provide a significant foundation for both basic and clinical research projects.

## Abbreviations

H & E: haematoxylin and eosin; IF: immunofluorescence; IHC: immunohistochemistry; TMA: tissue micro arrays.

## Competing interests

The authors declare that they have no competing interests.

## Authors' contributions

CK, MU and FP contributed to the conception and the supervision of the project. CK, JB, AA and FP wrote the manuscript. JB, MW, EL, SN and FP wrote the text corresponding to all normal, cancer and cell images. PO developed the database and website. All authors were involved in editing and the final approval of the paper.

## Authors' information

CK: associate professor and site Director for the tissue protein profiling facility, JB: PhD student (tissue biomarkers), PO: IT developer, AA: post-doctoral (immunohistochemistry-based cell profiling), SN: senior pathologist (immunohistochemistry-based tissue profiling), MW: PhD student (immunofluorescence-based cell profiling), EL: associate professor and responsible for the subcellular profiling unit, MU: professor and program Director for the Human Protein Atlas and FP: professor, senior pathologist and clinical Director for the Human Protein Atlas.

## Pre-publication history

The pre-publication history for this paper can be accessed here:

http://www.biomedcentral.com/1741-7015/10/103/prepub
